# Biosynthesis of the Essential Fatty Acid Oxidation Cofactor Carnitine Is Stimulated in Heart and Liver after a Single Bout of Exercise in Mice

**DOI:** 10.1155/2018/2785090

**Published:** 2018-05-29

**Authors:** Tom L. Broderick, Frank A. Cusimano, Chelsea Carlson, Jeganathan Ramesh Babu

**Affiliations:** ^1^Department of Physiology and Laboratory of Diabetes and Exercise Metabolism, Midwestern University, Glendale, AZ, USA; ^2^Department of Nutrition, Dietetics, and Hospitality Management, Auburn University, Auburn, AL, USA

## Abstract

We determined whether one single bout of exercise stimulates carnitine biosynthesis and carnitine uptake in liver and heart. Free carnitine (FC) in plasma was assayed using acetyltransferase and [^14^C]acetyl-CoA in Swiss Webster mice after 1 hour of moderate-intensity treadmill running or 4 hours and 8 hours into recovery. Liver and heart were removed under the same conditions for measurement of carnitine biosynthesis enzymes (liver butyrobetaine hydroxylase, γ-BBH; heart trimethyllysine dioxygenase, TMLD), organic cation transporter-2 (OCTN2, carnitine transporter), and liver peroxisome proliferator-activated receptor-alpha (PPAR*α*, transcription factor for *γ*-BBH and OCTN2 synthesis). In exercised mice, FC levels in plasma decreased while heart and liver OCTN2 protein expressed increased, reflecting active uptake of FC. During recovery, the rise in FC to control levels was associated with increased liver *γ*-BBH expression. Protein expression of PPAR*α* was stimulated in liver after exercise and during recovery. Interestingly, heart TMLD protein was also detected after exercise. Acute exercise stimulates carnitine uptake in liver and heart. The rapid return of FC levels in plasma after exercise indicates carnitine biosynthesis by liver is stimulated to establish carnitine homeostasis. Our results suggest that exercise may benefit patients with carnitine deficiency syndromes.

## 1. Introduction

Carnitine is an obligate cofactor that transfers long-chain fatty acids (FAs) into the mitochondria for the production of energy. Carnitine is derived from both dietary sources and endogenous biosynthesis [1]. The first reaction in carnitine biosynthesis involves the hydroxylation of trimethyllysine to 3-hydroxy-trimethyllysine by trimethyllysine dioxygenase (TMLD). Further enzymatic reactions involving a specific aldolase and a dehydrogenase (4-*N*-trimethylaminobutyraldehyde dehydrogenase, TMABA-DM) convert 3-hydroxy-trimethy-llysine to *γ*-butyrobetaine. In the final reaction, *γ*-butyrobetaine is hydroxylated by *γ*-butyrobetaine hydroxylase (*γ*-BBH) to form carnitine [2]. In the mouse, liver is the main site for synthesis and contains all the enzymes to produce carnitine, whereas heart and skeletal muscle lack *γ*-BBH. Therefore, *γ*-butyrobetaine is released from extrahepatic tissues into the plasma and accumulates in liver where it is hydroxylated to carnitine [3]. The uptake of carnitine from plasma by hepatic and extrahepatic tissues is facilitated by the sodium-dependent organic cation (OCTN2) transporter [4].

Carnitine biosynthesis is increased in conditions in which rates of FA oxidation are chronically elevated, such as caloric restriction, fasting, and high-fat feeding [5–7]. Under these conditions, activation of peroxisome proliferator-activated receptor-alpha (PPAR*α*) by nonesterified FAs (NEFAs) from adipose tissue increases the expression of *γ*-BBH and OCTN2 to facilitate the oxidation of FAs in tissues. FA oxidation is also increased in response to chronic exercise training, and similar to high fat feeding and caloric restriction, increases in the expression of *γ*-BBH and OCTN2 in rat liver have been reported [8]. However, the effects of acute exercise on *γ*-BBH and OCTN2 expression in liver remain to be determined, and since this enzyme is abundantly expressed in liver [2], it seems logical that exercise, by activating PPAR*α* through NEFAs, stimulates carnitine biosynthesis. Further, whether the heart participates in the initial step of carnitine biosynthesis or uptake of carnitine by this organ occurs with exercise is also not clear. Therefore, the purpose of this study was to investigate the effects of acute exercise on carnitine biosynthesis and uptake by liver and heart in the mouse.

## 2. Methods

### 2.1. Animals and Exercise Protocol

The Midwestern University Institutional Animal Care and Use Committee approved this study, and the animals used were cared for in accordance with the recommendations in the Guide for the Care and Use of Laboratory Animals (Institute of Laboratory Animal Resources 1996).

Male Swiss Webster mice were obtained at the age of 6 weeks (Charles River Laboratories, Wilmington, MA). After a one-week period of acclimatization, mice were randomly assigned to an exercise group or a control group. Mice in the exercise group were habituated to daily running on an electrically driven treadmill (Exer 3/6; Columbus Instruments, OH, USA) consisting of 5–10-minute sessions of running at 8–12 m/min for a period of 5 days, as previously described in our recent study [9]. Forty-eight hours after the final habituation exercise session, the acute exercise session was performed at a speed of 19 meters/min with an 8-degree grade for a period of 60 minutes. Based on treadmill belt velocity, the exercise intensity corresponds to ∼80% VO_2_ max of mice [10]. The 60-minute period included 10 minutes of warm-up gradually increasing the speed to 15 meters/min before the treadmill speed was set at 19 meters/min. Mice were euthanized immediately after exercise (acute group), at 4 hours after exercise (4-hour group), or 8 hours after exercise (8-hour group). Mice assigned to the control group were placed on a stationary treadmill adjacent to the treadmill used for the actual running sessions and euthanized at the same time points as exercising mice. In a separate group of mice (*n*=5), plasma was collected under identical exercise conditions for measurement of NEFA levels. Mice were housed 4 per cage and maintained in a room with a 12:12-hour light-dark cycle and fed a standard diet (Teklad, 18% protein diet, Harlan Laboratories, Madison, WI) and water ad libitum.

### 2.2. Tissue Harvest and Measurement of Plasma Metabolites

Mice were euthanized in an atmosphere of 100% CO_2_ followed immediately by surgical thoracotomy and inducing pneumothorax. Blood samples were obtained by cardiac puncture, and the plasma was separated by centrifugation and then stored at −80°C for subsequent measurement of carnitine by a radioenzymatic assay using carnitine acetyltransferase and [^14^C]acetyl-CoA [11]. NEFA levels in plasma were measured by calorimetry based on the acylation of coenzyme A (Wako Chemicals, Richmond, VA, USA). Liver and heart were removed, freeze-snapped with tongs cooled to the temperature of liquid N_2_, and stored for gene and protein expression.

### 2.3. Quantitative Real-Time Polymerase (PCR) Chain Reaction Analysis

RNA isolation and PCR methods have been previously described [8]. RNA content was isolated from liver using RNA STAT-60 following the protocol provided by the manufacturer (AMS Biotechnology, Abingdon, UK). First-strand cDNA was synthesized using the SuperScript^TM^ First-Strand Synthesis System (Invitrogen, Carlsbad, CA, USA). Quantitative real-time PCR was performed using primers listed in Table 1 with 18S rRNA (Sigma-Aldrich Life Science, The Woodlands, TX) used as the internal control. We chose 18S as an internal control to normalize cDNA input because this gene was not influenced by chronic exercise training [8, 12]. However, to confirm whether 18S was not influenced by acute exercise, *β*-actin and GAPDH were used as internal controls. No differences were observed with *β*-actin (*P*=0.3085) or GAPDH (*P*=0.6299) between control and exercise groups. Relative mRNA levels were quantified using the delta-delta CT method.

### 2.4. Western Blot Analysis

Analysis of protein content in liver and heart was performed using methods described previously [8]. Samples (20 *µ*g) were subjected to immunoblot analysis with the use of antibodies against OCTN2, *γ*-BBH, PPAR*α*, and TMLD (Santa Cruz Biotechnology Inc., Dallas, TX). As an internal control, blots were reprobed with an anti-actin antibody (Sigma-Aldrich Life Science, The Woodlands, TX). Values in the control group were normalized to arbitrary units for comparison with the exercise groups.

### 2.5. Statistical Analysis

Group mean differences were determined using a one-way analysis of variance, followed by a Tukey–Kramer comparison for post hoc analysis. All values are reported as the mean ± SEM. A value of *P* < 0.05 was considered statistically significant.

## 3. Results

There were no differences in body weight between groups of mice at the end of the study. Body weights for the control, acute, 4-hour, and 8-hour groups were 34.2 ± 0.2, 33.7 ± 0.8, 34.7 ± 0.8, and 35.1 ± 0.9 g, respectively. Because of similar body weights, heart and liver weights of mice from the three exercise groups were combined. Heart weight in the control and exercise groups was 0.138 ± 0.003 and 0.148 ± 0.005 g, respectively. Liver weight was 1.26 ± 0.10 and 1.42 ± 0.1 g, respectively, in control and exercising mice.

**Figure 1:**
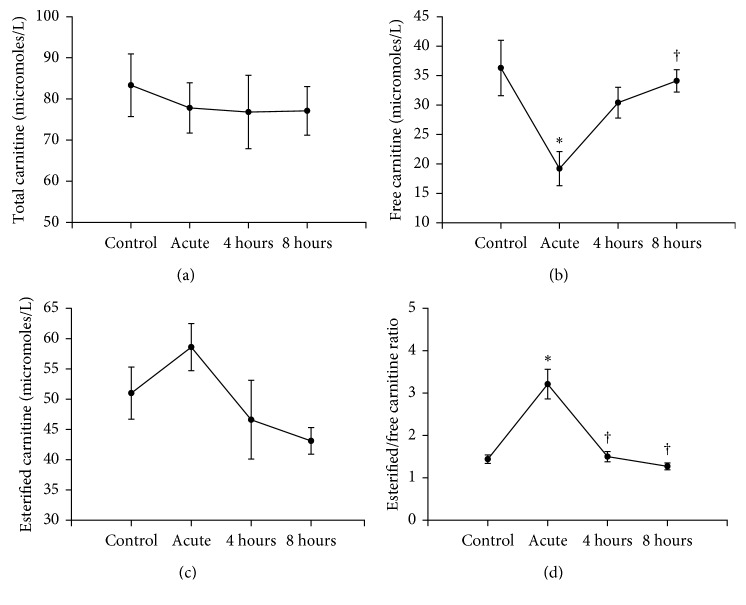
Effects of acute exercise on total (a), free (b), esterified (c), and esterified to free carnitine (d) levels in mouse. Plasma was obtained from mice immediately after exercise (acute) and 4 hours or 8 hours into recovery. Values are reported as mean ± SEM for 4 mice in each group. ^*∗*^*P* < 0.05 compared with control. ^†^*P* < 0.05 compared with the acute exercise group.

The effects of acute exercise and recovery on carnitine levels are shown in Figure 1. Levels of total carnitine, measured as the sum of free and esterified carnitine in plasma, remained unchanged throughout exercise and recovery (Figure 1(a)). However, acute exercise resulted in a significant decrease in the levels of free carnitine which was followed by a rapid return to control levels during recovery (Figure 1(b)). Plasma esterified levels were slightly increased during exercise, and recovery was marked by a return to control levels (Figure 1(c)). The esterified carnitine-to-free carnitine ratio was increased with exercise but decreased to control values during recovery (Figure 1(d)). The changes in free carnitine during exercise and recovery reflect uptake and biosynthesis of this cofactor, respectively.

**Figure 2:**
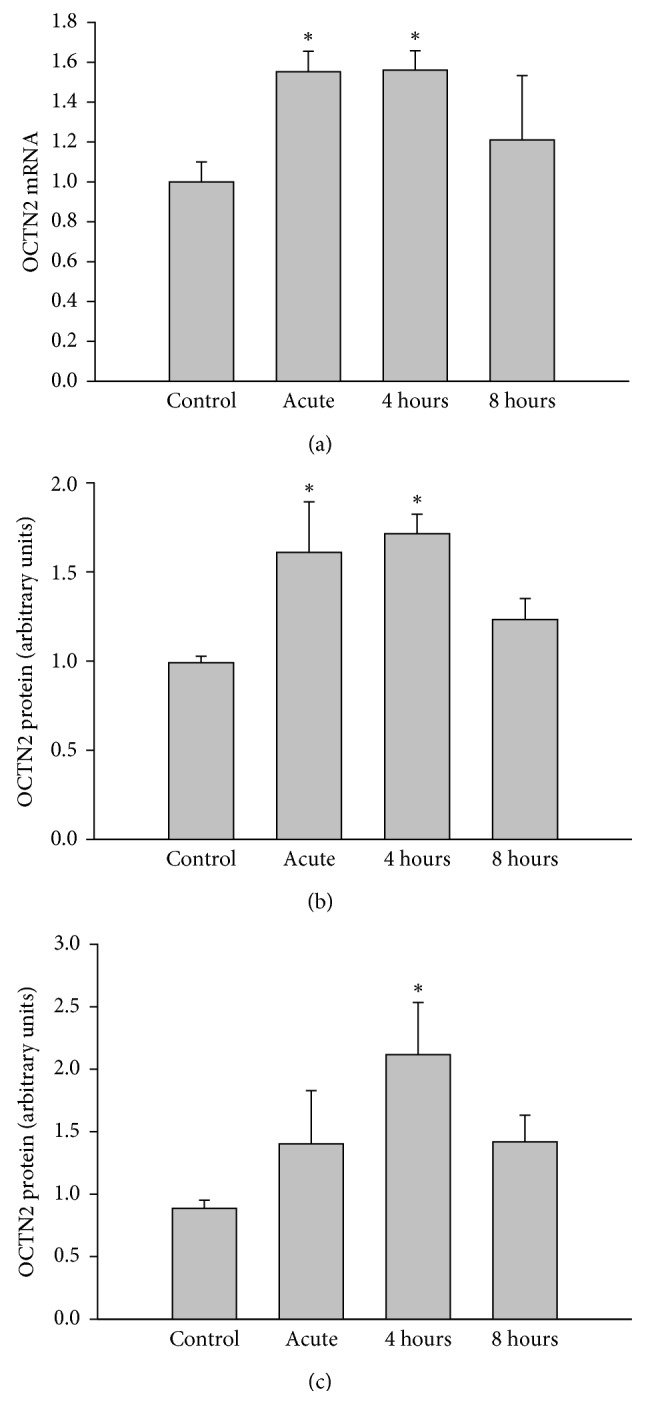
Effects of acute exercise on (a) liver mRNA OCTN2 expression (normalized for 18S), (b) liver protein expression of OCTN2 (normalized for actin), and (c) heart protein expression of OCTN2 (normalized for actin). Liver and heart were harvested from mice immediately after exercise (acute) and 4 hours or 8 hours into recovery. Values are reported as mean ± SEM for 8–10 mice in each group. OCTN2, organic cation transporter-2. ^*∗*^*P* < 0.05 compared with control.

**Figure 3:**
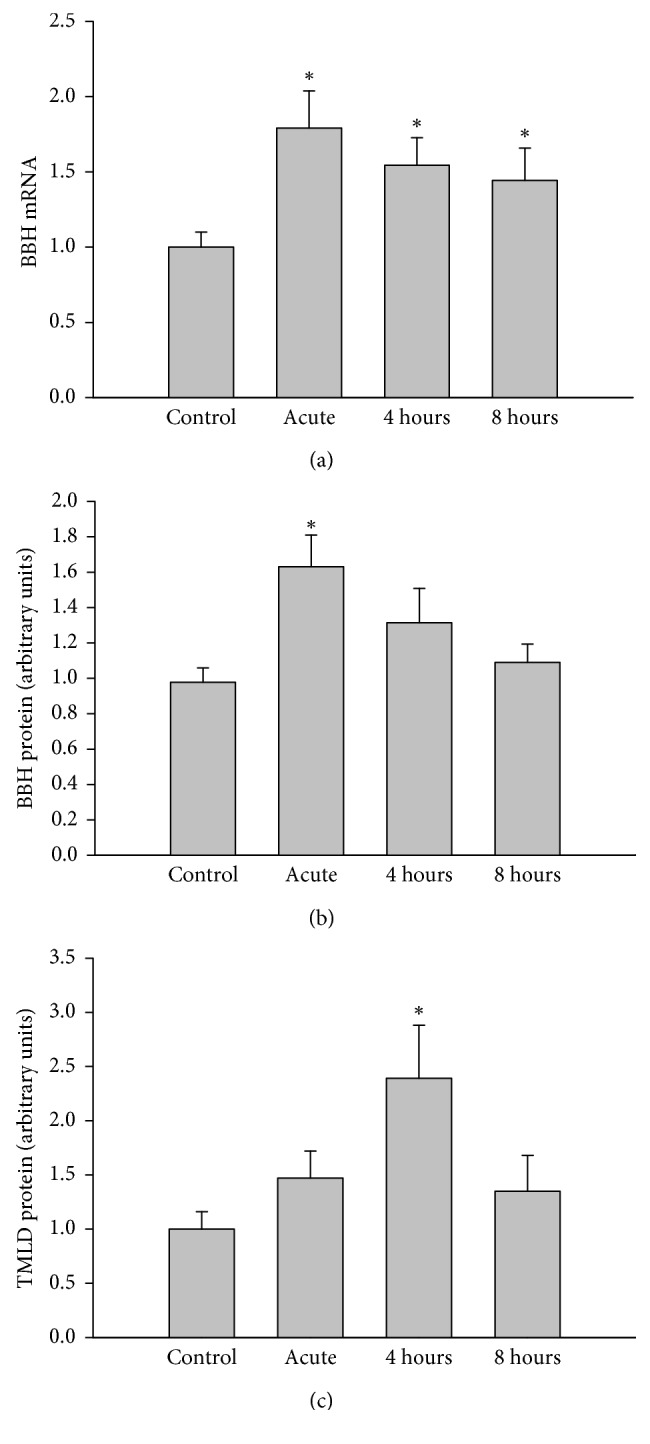
Effects of acute exercise on (a) liver mRNA *γ*-BBH expression (normalized for 18S), (b) liver protein *γ*-BBH expression (normalized for actin), and (c) heart protein TMDL expression (normalized for actin). Liver and heart were harvested from mice immediately after exercise (acute) and 4 hours or 8 hours into recovery. Values are reported as mean ± SEM for 8-9 mice in each group. BBH, *γ*-butyrobetaine hydroxylase; TMDL, trimethyllysine dioxygenase. ^*∗*^*P* < 0.05 compared with control.

To confirm that the decrease in free carnitine reflects active uptake, we determined the expression of OCTN2 in liver and heart. As shown in Figure 2, mRNA expression and protein expression of this transport protein were increased by ∼40% above control levels after exercise and recovery in liver (Figures 2(a) and 2(b)). In heart, a similar increase in protein expression was detected (Figure 2(c)). Carnitine biosynthesis is a function of multiple tissues with the last reaction involving the hydroxylation of *γ*-butyrobetaine to carnitine by *γ*-BBH occurring in liver. Heart also expresses the key enzymes of the biosynthetic pathway but lacks *γ*-BBH [3]. We determined whether the increase in the levels of free carnitine in plasma observed during recovery was associated with *γ*-BBH expression in liver. As illustrated in Figure 3, mRNA expression in liver was increased by ∼45% after exercise and during the recovery period (Figure 3(a)). This increase was met by a concomitant change in protein expression with exercise only (Figure 3(b)). We also found that TMLD, which converts trimethyllysine to 3-hydroxy-trimethyllysine, is also expressed following acute exercise in heart (Figure 3(c)).

**Figure 4:**
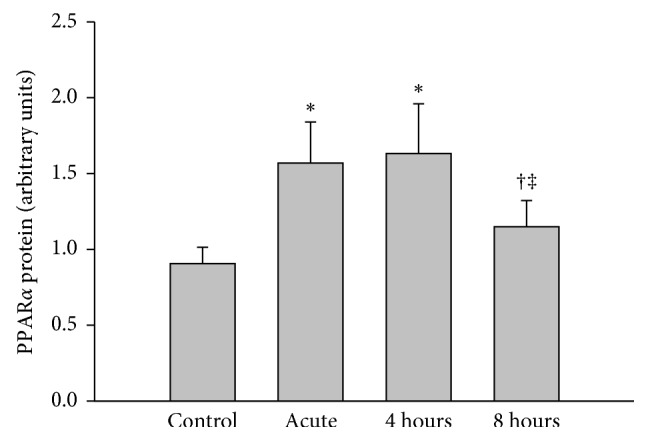
Effects of exercise on PPAR*α* protein (normalized for actin) expression in mouse liver. Liver was harvested from mice immediately after exercise (acute) and 4 hours or 8 hours into recovery. Values are reported as mean ± SEM for 8–10 mice in each group. PPAR*α*, peroxisome proliferator-activated receptor-alpha. ^*∗*^*P* < 0.05 compared with control. ^†^*P* < 0.05 compared with the acute exercise group. ^‡^*P* < 0.05 compared with the 4-hour group.

**Figure 5:**
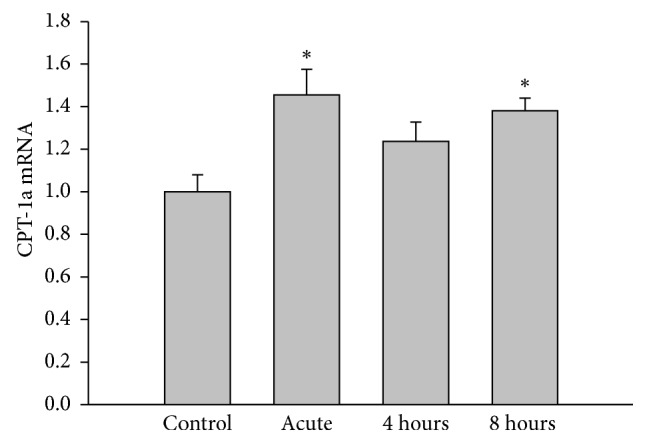
Effects of acute exercise on liver mRNA CPT-1a expression (normalized for 18S). Liver was harvested from mice immediately after exercise (acute) and 4 hours or 8 hours into recovery. Values are reported as mean ± SEM for 4 mice in each group. CPT-1a, carnitine palmitoyltransferase. ^*∗*^*P* < 0.05 compared with control.

Evidence shows that conditions associated with elevated rates of FA oxidation stimulate PPAR*α* expression in liver and genes involved in FA disposal, including *γ*-BBH and OCTN2 [7]. Whether acute exercise regulates PPAR*α* expression in liver is unclear. As shown in Figure 4, protein levels of PPAR*α* were increased in liver following exercise and remained elevated during the first 4 hours of recovery compared to the control group. To confirm whether FA oxidation was stimulated during exercise, mRNA expression of carnitine palmitoyltransferase 1a (CPT-1a) was measured in liver.  Figure 5 shows that mRNA expression of this enzyme was increased by ∼45% in response to acute exercise and remained elevated at 8 hours into the recovery period. In addition to the increase in CPT-1a expression in liver, the changes in plasma levels of NEFA also reflect an increase in FA oxidation induced by exercise. Although the levels of NEFA in plasma were not significant, a ∼17% decrease was observed after one hour of exercise (0.32 ± 0.02 in control versus 0.25 ± 0.02 mEq/L in acute group), indicating increased extraction of NEFAs by tissues. At 8 hours into recovery, however, plasma NEFA levels were nearly ∼80% higher compared to the control group (0.25 ± 0.02 versus 0.45 ± 0.16 mEq/L). Measurement of plasma NEFA 24 hours after exercise indicated a return to control levels (0.32 ± 0.02 versus 0.27 ± 0.01 mEq/L).

## 4. Discussion

The effects of acute exercise on carnitine biosynthesis and homeostasis are not well defined in the mouse. Our results indicate that acute exercise induced a significant reduction in the plasma levels of free carnitine while recovery was marked by a rapid return to control levels. These changes in plasma free carnitine indicate that both uptake mechanisms and biosynthesis, respectively, are activated. Indeed, increased protein expression of OCTN2 was detected in liver and heart from exercising mice. In liver, we observed an increase in the expression of *γ*-BBH, whereas in heart, TMDL expression was stimulated by acute exercise. Taken together, the changes in plasma free carnitine occurring with exercise and during recovery are consistent with uptake and biosynthesis. Further, considering the critical role of liver in carnitine biosynthesis [13], and the dramatic increase in protein levels of *γ*-BBH that persisted well into 4 hours into recovery, it is likely that return of carnitine homeostasis in plasma was attributed to output from the liver. Our results also highlight the effects of an acute metabolic stressor on carnitine homeostasis because most studies to date provide mechanisms in carnitine regulation that are reminiscent of chronic changes in FA oxidation [5–7]. Thus, both acute and chronic stressors activate carnitine uptake and biosynthesis to facilitate FA oxidation, but most remarkably, decreasing plasma free carnitine with exercise appears to be a robust stimulus. This raises the possibility of whether the disruption in plasma carnitine homeostasis from uptake by OCTN2 to enhance FA oxidation may serve as an initial stimulus to activate carnitine biosynthesis by tissues.

Early studies have indicated that carnitine biosynthesis and OCTN2 expression in liver are dependent on the activation of PPAR*α* [14], a lipid-sensing nuclear receptor that functions as a regulator of FA metabolism [15]. Evidence for this essential role of PPAR*α* on carnitine homeostasis was confirmed in hepatocytes overexpressing PPAR*α* and in PPAR*α*-null mice [16], the latter which mice developed carnitine deficiency as a result of decreased hepatic *γ*-BBH and OCTN2 expression [17]. Recent evidence indicates that transcriptional activation of TMABA-DM, which provides *γ*-butyrobetaine, and purportedly TMLD, is mediated by PPAR*α* [18]. The natural ligand for PPAR*α* activation is NEFA from adipose tissue, and with release of this ligand during acute exercise from lipolysis, the synthesis of genes relating to carnitine biosynthesis and uptake to support FA metabolism could be stimulated [19, 20]. In agreement with this mechanism, we demonstrated using the same exercise conditions that extraction of NEFAs from plasma was associated with PPAR*α* activation and increased expression of *γ*-BBH and OCTN2 in mouse kidney [9]. The recent observation that free carnitine content is increased in liver with exercise is consistent with this mechanism [21]. The increased esterified carnitine-to-free carnitine ratio in plasma and elevated mRNA CPT-1a content in liver reflect increases in fatty acid oxidation from exercise. The decrease in plasma NEFA induced by exercise indicates that this substrate was extracted from plasma and likely activated PPAR*α* and OCTN2 in tissues.

An interesting finding of this study was that acute exercise increased the expression of OCTN2 and TMLD in heart. Changes in OCTN2 are typically evident following chronic stress conditions, including fasting, caloric restriction, or exercise performed in the presence of diet-induced obesity [5–7]. Under these conditions, significant increases in the expression of this transporter occur to facilitate FA oxidation in tissues. In the present study, we show that acute exercise stimulated the expression for this carrier, an expected response considering the heart's oxidative nature and dependence on FAs as energy substrate particularly under exercise conditions when FA oxidation is elevated [22]. Indeed, contraction increases the translocation of intracellular stored FAT/CD36 to the plasma membrane followed by an increase in long-chain FA acid uptake into cardiomyocytes destined for mitochondrial *β*-oxidation [23]. The importance of carnitine on FA oxidation in heart is supported by studies examining training adaptations of which some include increases in mitochondrial density and free carnitine as well as improvements in endurance [24]. On the contrary, further supporting the role of carnitine on FA oxidation is the observation that healthy subjects and rats administered pivaloyl-conjugated antibiotics, known to induce severe systemic carnitine deficiency, demonstrate decreased heart rate and reduced rates of FA oxidation, decreased exercise capacity, and increased levels of ketone bodies in plasma [25, 26]. The expression of TMLD, the first in the biosynthetic carnitine pathway that converts trimethyllysine to 3-hydroxy-trimethyllysine, was also stimulated in response to acute exercise, an effect that remains to be confirmed by activation of PPAR*α* [18]. Increased expression of this hydroxylase in heart can potentially provide *γ*-butyrobetaine, the direct precursor of carnitine for final conversion to carnitine by liver or kidney [2]. Earlier studies have suggested that the availability of *γ*-butyrobetaine is considered to be a limiting step for biosynthesis [27], and increased serum *γ*-butyrobetaine levels following regular exercise in healthy subjects reflect a heightened supply of carnitine precursors from heart and skeletal muscle for carnitine biosynthesis [28].

In conclusion, we show that plasma carnitine homeostasis is disrupted with exercise but is rapidly restored in the mouse, indicating that carnitine biosynthesis mechanisms are stimulated. The return of FC in plasma during the recovery period is consistent with the increased *γ*-BBH expression in liver along with the possible indirect involvement of TMLD from heart. While exercise increases plasma levels of *γ*-butyrobetaine [28], and the OCTN2 transporter actively extracts this carnitine precursor from plasma, carnitine homeostasis is also achieved by OCTN2 expression in liver and heart although this will be addressed in our future studies. Further, because acute exercise can stimulate carnitine biosynthesis and uptake mechanisms, frequent exercise may benefit patients with primary and secondary carnitine deficiency syndromes.

## Figures and Tables

**Table 1:** Real-time polymerase chain reaction primers.

Gene product	Primer sequences
*γ*-BBH	Forward primer: AGCAGGACTCCAAGGAGAAT
	Reverse primer: CACAAAGGTGGAGGAGAGAA

OCTN2	Forward primer: GGGCTACTTCGGACTTTCTC
	Reverse primer: GGGAAGAACAAGGTGAGGAT

CPT-1a	Forward primer: CCAGTCTCAGCCTCTACGGC
	Reverse primer: CACAGTGTCCTGTCTCCGTG

18S rRNA	Forward primer: AGTCCCTGCCCTTTGTACACA
	Reverse primer: CGATCCGAGGGCCTCACTA

BBH, *γ*-butyrobetaine hydroxylase; OCTN, organic cation transporter; CPT-1a, carnitine palmitoyltransferase isoform 1a. Reproduced from Broderick et al. [9] (under the Creative Commons Attribution License/public domain).
